# Shooting at a Moving Target—Effectiveness and Emerging Challenges for SARS-CoV-2 Vaccine Development

**DOI:** 10.3390/vaccines9101052

**Published:** 2021-09-22

**Authors:** Franziska Günl, Angeles Mecate-Zambrano, Selina Rehländer, Saskia Hinse, Stephan Ludwig, Linda Brunotte

**Affiliations:** 1 Institute of Virology (IVM), University of Münster, 48149 Münster, Germany; guenl@uni-muenster.de (F.G.); a_meca01@uni-muenster.de (A.M.-Z.); s_rehl01@uni-muenster.de (S.R.); s_hins05@uni-muenster.de (S.H.); ludwigs@uni-muenster.de (S.L.); 2 Interdisciplinary Centre for Clinical Research (IZKF), Medical Faculty, University of Münster, 48149 Münster, Germany

**Keywords:** SARS-CoV-2, vaccines, variants, vaccine safety, vaccine effectiveness, adverse effects, heterologous vaccination, breakthrough infection, long Covid, second generation vaccines

## Abstract

Since late 2019 the newly emerged pandemic SARS-CoV-2, the causative agent of COVID-19, has hit the world with recurring waves of infections necessitating the global implementation of non-pharmaceutical interventions, including strict social distancing rules, the wearing of masks and the isolation of infected individuals in order to restrict virus transmissions and prevent the breakdown of our healthcare systems. These measures are not only challenging on an economic level but also have a strong impact on social lifestyles. Using traditional and novel technologies, highly efficient vaccines against SARS-CoV-2 were developed and underwent rapid clinical evaluation and approval to accelerate the immunization of the world population, aiming to end the pandemic and return to normality. However, the emergence of virus variants with improved transmission, enhanced fitness and partial immune escape from the first generation of vaccines poses new challenges, which are currently being addressed by scientists and pharmaceutical companies all over the world. In this ongoing pandemic, the evaluation of SARS-CoV-2 vaccines underlies diverse unpredictable dynamics, posed by the first broad application of the mRNA vaccine technology and their compliance, the occurrence of unexpected side effects and the rapid emergence of variations in the viral antigen. However, despite these hurdles, we conclude that the available SARS-CoV-2 vaccines are very safe and efficiently protect from severe COVID-19 and are thereby the most powerful tools to prevent further harm to our healthcare systems, economics and individual lives. This review summarizes the unprecedented pathways of vaccine development and approval during the ongoing SARS-CoV-2 pandemic. We focus on the real-world effectiveness and unexpected positive and negative side effects of the available vaccines and summarize the timeline of the applied adaptations to the recommended vaccination strategies in the light of emerging virus variants. Finally, we highlight upcoming strategies to improve the next generations of SARS-CoV-2 vaccines.

## 1. Origin and Evolution of SARS-CoV-2 in Humans

SARS-CoV-2 is a member of severe acute respiratory syndrome-related coronaviruses that belongs to the betacoronavirus genus (subgenus: sarbecovirus). This genus also includes the seasonal common cold-causing HCoV-OC43 and HCoV-HKU1 strains as well as SARS-CoV and MERS-CoV, the causative agents of previous epidemics in China (2003) and Saudi Arabia (2012), respectively. The large positive sensed, single-stranded RNA genome with a size of around 30 kb encodes for approximately 14 open reading frames (ORFs) [1]. The genome sequence of SARS-CoV-2 suggests a close relation to the sarbecovirus genomes RaTG13 and RmYN02 from bats [2,3]. Considering the mechanism of genomic recombination that occurs in coronavirus genomes, several other bat-derived virus strains demonstrate high sequence homology to parts of the SARS-CoV-2 genome, suggestive of a shared common coronavirus ancestor [4,5,6]. The high similarity of coronavirus genomes from other animals also indicates the involvement of intermediate hosts in the evolution and zoonotic transmission of SARS-CoV-2 to humans [7,8]. However, also in humans, SARS-CoV-2 continues to evolve, and new variants with mutations, primarily located in the surface-exposed spike (S) protein, have emerged over time with strong impacts on the real-world effectiveness of vaccines.

**Figure 1:**
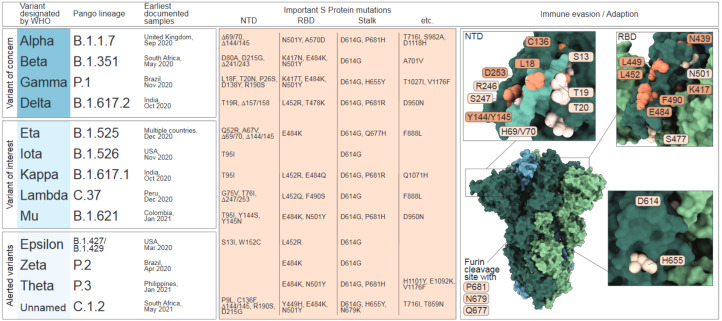
Table of Variants characterized as Variant of concern (VOC), Variant of interest (VOI) or Alerted Variant by the World Health Organization (WHO). Left panel: shown is the Greek letter nomenclature as introduced by May 2021, the Pango nomenclature together with the date and location of the earliest documentation of a detected sample of the respective variant. Middle panel: List of the characterizing mutations based on their location in the N-terminal domain (NTD), receptor binding domain (RBD) or stalk region. Right panel: The crystal structure of the Spike protein trimer in closed conformation (PDB: 6ZGI) and an illustration of the mutated residues based on their location in the NTD, RBD or stalk region. Mutations that have been reported to confer immune escape are highlighted in orange. NTD zoom in: the NTD supersite, as described by McCallum et al. [12], is highlighted in lighter green.

Since the first reported emergence in Wuhan City, Hubei Province, China, in December 2019 extensive genome sequencing and data sharing have enabled tracing of SARS-CoV-2 outbreaks and global spreading, as well as the real-time detection of mutations in the viral genome that led to the emergence of new SARS-CoV-2 variants [2,9,10]. While the majority of SARS-CoV-2 genome variation reflects synonymous or transient mutations with limited biological impact, several mutations are now, based on scientific evidence, associated with human adaptation and immune escape (reviewed in [11]). To prioritize variants with respect to their public health relevance, the World Health Organization (WHO) has defined variants of interest (VOI) and variants of concern (VOC). In contrast to VOIs with locally restricted spreading patterns, VOCs demonstrate increased transmission, virulence, pathogenicity or a reduced susceptibility to public health measures, diagnostics, vaccines or therapeutics and present a dominantly spreading phenotype. To date, the WHO has classified four lineages as VOC, which include Alpha (B.1.1.7.), Beta (B.1.351), Gamma (B.1.1.28.1; in the following referred to as P.1) and Delta (B.1.617.2). Five lineages were classified as VOI, which include Eta (B.1.525), Iota (B.1.526), Kappa (B.1.617.1), Lambda (C.37) and Mu (B.1.621). Moreover, the WHO lists multiple lineages with the alert for further monitoring, including the three former VOIs Epsilon (B.1.427/B.1.429), Zeta (B.1.1.28.2; in the following referred to as P.2) and Theta (B.1.1.28.3; in the following referred to as P.3) as well as the recently listed C.1.2 variant (Figure 1).

The determinants for VOC/VOI classification are amino acid (AA) variations in the S protein, which facilitates binding to the cellular receptor angiotensin converting enzyme 2 (ACE2) and is exposed as trimers on the viral surface. Each trimer is composed of a variable globular head that comprises the N-terminal domain (NTD; AA 13-305) and the receptor-binding domain (RBD; AA 319-541) as well as a conserved stalk region, which harbors the cleavage sites for host proteases, such as furin and transmembrane serine protease 2 (TMPRSS2). As the receptor-binding entity and the major target for neutralizing antibodies (nABs), the S protein plays a pivotal role in transmissibility, infectivity and immunity [13,14,15,16,17].

The first stable mutation in the SARS-CoV-2 genome, a change at position 614 in the stalk region of the spike protein (D614G), was reported in early 2020, establishing the new Newstrain G clade or Pango Lineage B.1. The location of this mutation within a highly conserved region of the S protein and its independent emergence in several locations indicated human-host adaption (Figure 1, lower panel) [1]. Demonstrating a dominant spreading phenotype, this mutation rapidly outcompeted previously circulating SARS-CoV-2 strains on a global scale, which corroborates its host adaptive function [18,19,20]. Mechanistically, this mutation is associated with structural improvements that increase the binding to ACE2 by stabilizing the RBD in the receptor-accessible UP-position [21,22,23,24]. Hence, this variant demonstrated increased infectivity and replication in cells and animal models, enhanced transmission in transgenic mice and hamsters, and reached higher viral loads in upper respiratory tracts of infected hamsters and human individuals [11,19,21,25,26,27,28,29]. However, in epidemiologic studies, an association of D614G with higher virulence or clinical severity could not be established [18,19]. Studies that assessed changes in immunity demonstrated that stabilized RBD UP-conformation of D614G variants also increases epitope exposure, which leads to an enhanced neutralization by monoclonal antibodies (mAbs) or sera of either infected or vaccinated patients [23,24,26,27].

One position that gained increased attention due to its mutation in VOCs with dominant spreading phenotypes, was P681. In the Alpha, Delta and Theta variants P681 is substituted with arginine (R) or histidine (H), which leads to an extension of the furin cleavage site and thereby increases furin-mediated cleavage, which has been suggested to contribute to faster replication, increased transmission and pathogenicity [30,31,32,33,34,35,36]. Experimentally, P681R/H substitution was reported to enhance cell-cell fusion and escape from nABs, despite decreased viral infectivity [31,36,37]. Due to their close proximity to the furin cleavage site, both the Q677H and the N679K mutation, that emerged in the Eta and C.1.2. variant, respectively, might be also associated to increased cleavage [38]. Moreover, the mutually exclusive emergence of either N679K or P681H mutations in a portion of Gamma and C.1.2. variant isolates support the hypothesis of functional resemblance [39]. H655Y, also detected in Gamma isolates, has not been functionally characterized yet.

As the most exposed region, the RBD is targeted by approximately 90% of the nABs in COVID-19 convalescent sera and has been subject to multiple mutations that contribute to human adaption, viral fitness and immune escape [40,41,42,43,44,45,46,47,48]. A key mutation that is shared by the Alpha, Beta, Gamma, Theta, Mu and C.1.2 variants is N501Y, which contributes to a substantial transmission advantage of these variants mediated by increased binding affinity to the ACE2 receptor [18,49,50,51,52,53,54,55,56,57,58]. However, studies using sera of BNT162b2-immunized patients demonstrated no reduced susceptibility to the neutralization of S proteins with only the N501Y SNP [59,60]. Additional mutations in the RBD with high global prevalence are N439K, L452Q, S477N and E484K, which also enhance ACE2 binding and, in case of N439K, increase viral loads [56,57,58,61]. The substitution K417N/T, present in the Beta and Gamma variants, is reported to reduce the receptor interaction by disruption of a salt bridge formed by K417 with D30 in ACE2 [56,57]. In nature, however, substitutions of K417 mostly occur in combination with N501Y and E484K, which appear to rescue the interaction with ACE2 [62].

Besides affecting the interaction with ACE2, RBD mutations K417N, N439K and F490S also contribute to immune escape (Figure 1, upper right panel) [44,45,57,61,62,63,64,65,66,67]. Interestingly, as in case of the K417N mutation, the substitution of Y449 to a histidine, which recently emerged on the RBD surface of C. 1.2 variants has also been reported to reduce ACE2 receptor binding but confers immune escape to certain class 1 and class 3 antibodies [56,64,65,67]. However, the best characterized immune escape mutation resides at position E484K/Q. This mutation independently emerged worldwide and is prevalent in most variants, including Beta, Gamma, Delta, Zeta, Eta, Iota, C.1.2 and also in several isolates of the B.1.1.7 lineage (denoted as B.1.1.7+E484K) [68,69]. E484K is majorly responsible for reduced neutralization by either infection-induced antibodies or commercially available mAbs, such as Bamlanivimab that is used for COVID-19 treatments in immune suppressed patients and known to induce the emergence of E484K [40,41,42,43,44,45,46,48,64,65,66,67,70,71].

Due to its occurrence in several highly transmissible variants, the RBD mutation L452R/Q has recently gained much attention. It was reported to increase viral infectivity and spread and to confer partial immune escape [44,64,65,66,69,72,73]. Substitution of the adjacent position Y453F was previously observed in a virus cluster from minks in mid-2020 (lineage B.1.1.298) and displayed increased interaction with both mink and human ACE2 receptors [35,56,74,75]. Interestingly, the substitution of L452 with glutamine, present in the Lambda variant, but not with arginine as present in Delta, Epsilon and Eta, contributes to enhanced ACE2 binding [56].

Sequence analyses of the S protein revealed that the NTD is the least conserved domain and contains multiple key mutations shared by several variants [61]. Intriguingly, these mutations are also associated with immune escape, suggesting that, like the RBD, the NTD is under constant selective pressure driven by the host humoral immune response [76]. Reports that identified the accumulation of amino substitutions located in the NTD in immunocompromised patients with prolonged infections support this theory [12,16,77]. Recent reports find that, despite the bold glycan modification, which shields immunogenic epitopes of NTD, 10–20% of the neutralizing antibodies in convalescent sera are directed against this domain, implicating that the NTD is a driving factor for the development of immune escape [78,79,80,81]. Indeed, the most prevalent mutations in the NTD are surface-exposed and located in antigenically significant regions [12]. One of these regions harbors the glycosylation site N17, which clusters with mutations S13I (Epsilon), L18F (Beta, Gamma), T19R (Delta) and T20N (Gamma) (Figure 1). Notably, L18F abrogates neutralization by mAbs directed against the NTD. The same applies for mutations located in an immunodominant loop in close proximity to the N149 glycosylation site, which includes W152C (Epsilon), C136F (C1.2) and D138Y (Gamma) as well as Y144S and Y145N (Mu). The deletion of Y144/Y145 (referred to as del144) is prevalent in Alpha, Iota and C.1.2 variants and further emerged during experimental infection of Syrian hamsters treated with mAbs targeting the NTD [12]. The third antigenic region clusters around a loop that spans AA 245-265. Mutations in this region were identified in the Beta (del241/243, R246I), Iota (D253G) and Lambda (del247/253) variants. Notably, substitutions at position D253 contributed to both, escape from mAbs and reduced susceptibility to neutralization by NTD-directed mAbs [80]. The broadly discussed deletion of positions H69/V70, which is found in the Alpha variant and also independently occurred in some isolates of other VOCs and VOIs, leads to increased infectivity mediated by enhanced spike cleavage [82]. However, this deletion has not been shown to substantially contribute to escape from neutralization by mAbs [59,60,83].

## 2. Clinical Evaluation and Real-World Effectiveness of First-Generation SARS-CoV-2 Vaccines

The first generation of vaccine prototypes against SARS-CoV-2 are based on the S protein amino acid sequence of the early Wuhan-Hu-1 (Nextstrain: clade A) isolate. Vaccine approaches included various technologies, ranging from inactivated viruses to novel technologies that employ nanoparticle-based strategies or modified mRNA. This special design of the nucleic acid leading to altered amino acids in the S protein is meant to increase vaccine antigenicity. For example, both mRNA-based vaccines developed and provided by Biontech/Pfizer and Moderna, include a di-proline at residues 986 and 987 in the S2 subunit, which has been shown to prevent premature S1 shedding, a process that was first shown in MERS [84,85]. With the same intention, Janssen/Johnson&Johnson (J&J) and Novavax developed vaccines based on S proteins lacking the furin cleavage site [86,87].

Commonly, new vaccine candidates for infectious diseases undergo a stringent multiphasic clinical assessment procedure in which each phase aims to evaluate a specific aspect of the vaccine. Early phase I trials aim to assess safety and optimal dosage using small groups of healthy volunteers (20 to 80 individuals). Phase II aims to evaluate the immunogenicity generated by the vaccine, employing cohorts of hundreds of persons with characteristics that resemble the target group of the vaccine that include age, sex, comorbidities or a specific disease. In phase III trials, data from large cohorts of recruited participants belonging to different subgroups of age, sex, ethnicity and comorbidities are used to confirm vaccine safety, which includes intensity of expected reactions (reactogenicity) and adverse effects, and most importantly, to establish overall vaccine efficacy. Phase III trials are usually multi-centric and take place at locations with high infection incidence under strictly controlled conditions. During health emergencies like the current COVID-19 pandemic, outcomes of phase III trials can be submitted to regulatory entities like the Federal Drug Administration (FDA) to obtain emergency use authorization (EUA) while further clinical investigations are still ongoing. For SARS-CoV-2 vaccines, the endpoints of phase III trials evaluated the efficacy of a specific vaccination regimen to prevent severe disease, hospitalization and death by comparison of a vaccinated test group with a placebo control cohort. Other important criteria, such as the efficacy of protection from viral infection and virus shedding are less frequently evaluated and remain to be determined in real-world settings. As vaccine efficacy and safety underlies many factors that are rigorously controlled in clinical trials, the “real-world” effectiveness of a vaccine in a natural population undergoes final evaluation during long-term phase IV trials or epidemiological studies that are performed in parallel to vaccine roll out and aim to evaluate vaccine effectiveness and potential long-term side effects.

### 2.1. mRNA Vaccines

mRNA based vaccines represent a novel technology that has been developed and widely characterized for many years prior to its application for SARS-CoV-2 vaccines. To induce an antigen-specific immune response, these vaccines utilize mRNA molecules that encode the sequence of the SARS-CoV-2 spike protein and are packaged in lipid vesicles as carriers. Upon injection, the mRNA molecules give rise to S proteins that are displayed on dendritic cells and presented to immune cells. This stimulates potent humoral immune responses mediated by antigen-specific IgG and IgA antibodies released from differentiated circulating B cells that generate high levels of these antibodies in the blood and the respiratory mucosa [88,89,90,91]. In addition to serving as templates for cellular translation, mRNA molecules act as potent stimulators of the host innate immune response that is required to achieve optimal and long-lasting protection. This is facilitated by the recognition of the mRNA molecules by cellular RNA sensors, such as TLR3 and TLR7, MDA5, RIG-I, NOD2 and PKR that leads to the secretion of type I interferons and inflammatory mediators, which are needed for the recruitment and differentiation of naïve T cells (reviewed in [92]). Indeed, several studies have demonstrated that S protein-specific CD8^+^ T effector cells were detected already only one week after vaccination. Furthermore, CD4^+^ T helper and memory cells, which ensure long-term protection could be detected up to six months later [93,94,95,96,97,98,99].

Despite deployment hesitation due to potential low stability, lipid formulation and lack of previous human usage, mRNA vaccines have convincingly demonstrated to induce robust humoral responses and extraordinary efficacies and were the first to receive EUA [100,101].

#### 2.1.1. BNT162b2 (Biontech/Pfizer)

**Figure 2:**
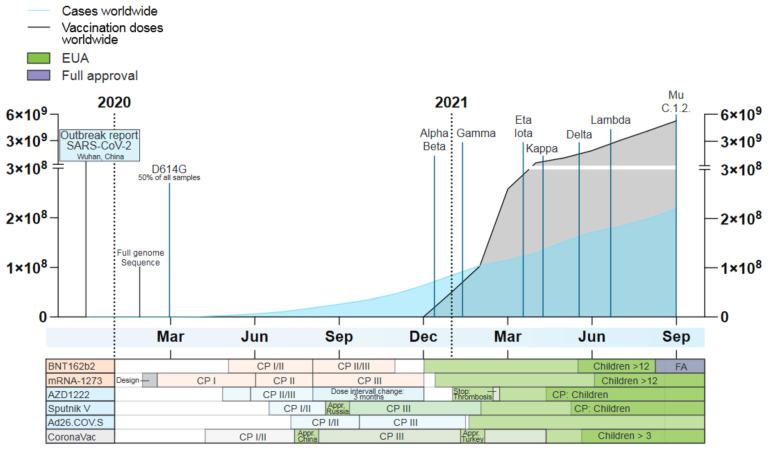
Timeline of the number of worldwide detected SARS-COV-2 cases (blue) and administered vaccination doses (grey), the time point of designation as a VOC or VOI by the WHO as well as key dates during the development of currently approved vaccines based on their technology: mRNA vaccines (orange), adenovirus-based vaccines (blue) and recombinant protein vaccines (grey). Given is the timeframe from the outbreak report of SARS-CoV-2 by the WHO in December 2019 until September 2021. Data taken from ‘Our world in data’ (September 2021) [104]. CP = clinical phase, Appr. = Approval, EUA = emergency use authorization, FA = Full approval.

The first SARS-CoV-2 vaccine receiving FDA authorization on 11 December 2020, was BNT162b2 from Biontech/Pfizer (Comirnaty) (Figure 2). Early that year the company had submitted two different vaccine designs for evaluation in phase II trials: BNT162b1, encoding only the RBD-Spike domain, and BNT162b2 encoding the full length-spike protein. Findings of this trial showed that BNT162b2 induced lower incidence of systematic adverse reaction in the participants, especially in older adults and thus, only BNT162b2 proceeded to the next phase [102]. Phase III trials with BNT162b2 demonstrated an efficacy of 94% among all tested age groups (18–55 and >55 years old) applying a two-dose regimen, three weeks apart (ClinicalTrials.gov: NCT04368728). Only mild adverse reactions to the vaccine were recorded. Endpoints of the study were focused on prevention of severe infection and hospitalization. Groups below 55 years of age reported a higher frequency of intense pain, fever and fatigue upon the second dose compared to older groups [103].

After EUA, mass vaccination programs have reached millions of individuals worldwide and follow-up studies have started to picture the “real-world” effectiveness of BNT162b2. First data from Israel, where more than 50% of the population is fully vaccinated with BNT162b2, has reported vaccine effectiveness of 97% against symptomatic cases. This was accompanied by a reduction of 97.2% of hospitalization related to infection, a 97.5% reduction in severe disease, a 96.7% reduction in fatal cases and, of note, a 91.5% reduction in asymptomatic infections [105]. In addition, a reduction of 50–70% in the incidence of PCR-confirmed SARS-CoV-2 infections upon the first dose was reported [106]. Recently, a study from more than 800,000 fully vaccinated individuals in southern Sweden, demonstrated 86% of effectiveness in preventing COVID-19. This was observed in the age groups from 16–64 years old with no differences between male and female individuals [107]. In addition, BNT162b2 vaccination effectiveness in vulnerable groups like elderly was evaluated in Finland. With a cohort of more than 900,000 subjects over 70 years old, full vaccination granted a 75% protection against symptomatic disease and protection of 93% against hospitalization due to COVID-19 [108]. All this data suggests that BNT162b2 is a safe and efficacious vaccine, including for more vulnerable age groups. Hence, full approval was granted by the FDA in August 2021.

#### 2.1.2. mRNA-1273 (Moderna)

The mRNA-1273 vaccine from ModernaTX received EUA on 18 December 2020, one week after approval of BNT162b2. Similar to BNT162b2, this vaccine was able to protect from severe COVID-19 at a calculated efficacy of 95% using a two-dose regimen, 28 days apart, as observed in the phase III trial (ClinicalTrials.gov: NCT04470427). The robust protection was observed consistently across all assessed age subgroups (18–65 years old) [109]. Delayed local skin and muscle reactions several days after injection have been reported as post-trial side effects. However, as these reactions resolved within six days, they were not a reason for concern [110]. A prospective study of vaccinated medical personal and frontline workers in the USA has estimated a vaccine effectiveness of 90% in reducing infection, regardless of the symptom status, as confirmed by real-time (RT-) PCR, 14 days after full immunization in a cohort vaccinated with BNT162b2 (62.7% of total population) and mRNA-1273 (29.6%) [111]. However, reports on the effectiveness in the general population are still awaited.

#### 2.1.3. CVnCoV (CureVac)

As a third mRNA vaccine provider that reached phase III trials, the company CureVac announced the results of their combined phase IIb/III trial with the first-generation SARS-CoV-2 vaccine candidate, CVnCoV, in June 2021. In contrast to the other two mRNA vaccines, which employed mRNA with modified nucleosides [112], CVnCoV is based on unmodified mRNA that encodes a full-length, pre-fusion stabilized S protein.

A multi-centered study (ClinicalTrials.gov: NCT04652102) in ten different countries in Latin America and Europe demonstrated an overall vaccine efficacy of 48% against COVID-19 of any severity, 77% against severe disease and 100% in reducing hospitalization and death for the groups 18–60 years old; in the group whose population was over 60 years old, pre-established statistical success criteria were not met. The sequencing of 204 positive cases from this study in different countries demonstrated that 51% of the sequences belonged to SARS-CoV-2 VOCs, although the lineages were not completely specified. The company highlighted that 21% of the sequences corresponded to the Lambda variant, which was recently upgraded to a VOI by the WHO due to its increased-transmission phenotype and the presence of several mutations in the S protein that may confer partial escape from the vaccine-induced antibodies. The company claimed that the reduced efficacy was due to the unprecedented broad diversity of 15 variants that were endemic in the study locations. The official version of this study is yet to be published.

#### 2.1.4. Effectiveness of mRNA Vaccines against Variants

Since the recognition of the emerging SARS-CoV-2 VOCs and VOIs, vaccine companies, as well as the scientific community, rapidly initiated studies to evaluate the potential of first-generation vaccines to neutralize these emerging variants. In parallel, epidemiological studies were focused on estimating the effectiveness against these variants during the ongoing vaccination rollout worldwide.

These studies demonstrate that the effectiveness of mRNA vaccines against the Alpha variant appears to be high for the first-generation vaccine design. Early evaluation of human serum samples following full vaccination with BNT162b2 demonstrated similar neutralizing antibody titers against the Alpha variant compared to the ancestral strain USA-WA1/2020 [113]. Importantly, this is largely reflected in the real-world data from mass vaccination programs in different countries. In Qatar, where 50% of the total COVID-19 cases corresponded to the Alpha variant, data showed an 89.7% effectiveness in reducing symptomatic infection and 97.4% in preventing severe disease, hospitalization or fatal cases [114]. In Israel, where more than 80% of the cases are accounted to this strain, vaccine effectiveness reached 92% against infection, 92% against severe disease and 87% against hospitalization [106]. In the UK BNT162b2 reached 93.7% effectiveness against symptomatic COVID-19 [115], and in Canada a high effectiveness of 89% against this variant was reported [116]. A nationwide study in France confirmed the high effectiveness of this vaccine observed in other countries, demonstrating an 86% effectiveness after seven days from the second dose, 83% within two to six months after vaccination and 84% after six months of vaccination against any type of infection severity [117]. Similar to BNT162b2, Moderna assessed the level of protection of their mRNA vaccine mRNA-1273 against the Alpha variant, demonstrating efficiently neutralized infection of VSV pseudotyped viruses encoding the S protein of this VOC in serum samples from fully vaccinated individuals [118]. Real-world data from Qatar estimated an effectiveness of mRNA-1273 against symptomatic disease of 100% and severe disease, hospitalization and death, an efficacy of 95.7% [119]. Consistently, Canada observed an effectiveness of 92% for this strain against symptomatic disease [116].

The protection generated against the Beta and Gamma variants seems to be insufficient with the first generation of mRNA vaccines. Neutralizing activity of serum samples from vaccinees demonstrated lower neutralization titers (two-fold) with BNT162b2 when challenged with VSV viruses carrying amino acid changes for the Beta variant in comparison with the control strain USA-WA1/2020 [113]. Consequently, the company evaluates the effects of a third dose and a second-generation vaccine based on the Beta variant S protein, BNT162b2SA, in order to enhance the protection, specifically, for this VOC (ClinicalTrials.gov Identifier: NCT04368728). Moderna also observed a decrease in neutralization for the Beta variant (6.4-fold) in serum samples that were evaluated by plaque-reduction neutralization compared to the control virus [118]. However, data from mass vaccination has not reflected this apparent waning protection. Data from Qatar, where 44.5% of the total analyzed infection corresponded to this VOC, estimated an effectiveness with BNT162b2 vaccination of 75% against symptomatic disease and 97.4% against more severe forms of the infection [114]. Of interest, effectiveness of mRNA-1273 in this country was later reported to be 96.4% against symptomatic infection and 95.7% against hospitalization, severe infection and death [119]. Consistently, Canada has reported protection estimated at 84% against Beta/Gamma infections, independently of the severity of infection upon BNT162b2 immunization [116]. France, in their nationwide study, reported a 77% protection against Beta/Gamma infections one week after vaccination and a slight decrease in effectiveness of 74%, six months after vaccination [117].

Vaccination effectiveness against the Delta variant, the predominant variant in several countries including the US, UK and Scotland until July 2021, seems to be preserved against severe forms of infection, hospitalization and preventing death. However, for symptomatic disease protection of mRNA vaccines is reduced, at least in the US, as reported by the Centers for Disease Control and Prevention (CDC). From 3 May to 25 July 2021, the predominance of this VOC raised from 2% to 80% in this country. The overall age-adjusted effectiveness against new cases for all adults declined from 91.7% to 79.8%, whereas during the same period, the effectiveness against hospitalization remained steady, ranging from 91.9% to 95.3% [120]. This suggests limited protection with the current vaccines. Previous to this study, Biontech/Pfizer had reported slightly reduced neutralizing titers of BNT162b2 immunization against the Delta variant in serum samples in comparison with the reference strain USA-WA1/2020 (log_2_PRNT_50_ 355 vs. 502). Of note, in the same study, the Delta-related strain Kappa that carries the immune escape mutations RBD-E484Q presented the strongest decrease in titers (log_2_PRNT_50_ 157) [113]. Supporting these observations, Moderna reported that the virus-neutralizing properties of sera samples from eight fully vaccinated individuals were reduced by 2.1-fold for Delta and 3.4-fold for Kappa in comparison with the reference isolate [121], evincing the necessity of a more meticulous monitoring of the Kappa variant, currently labeled as VOI. Interestingly, these results observed in sera are not reflected in vaccination effectiveness.

The UK has claimed an effectiveness with BNT162b2 vaccination of 88% against symptomatic COVID-19 infections by this VOC [115]. Supporting this, Scotland has observed protection rate of 92% against any level of infection by the Delta variant upon BNT162b2 vaccination [122]. Most of the reviewed studies report data for adult age groups. However, effectiveness in elderly groups seemed to be even more reduced against this variant. The CDC has reported vaccine effectiveness for mRNA vaccines (Biontech/Pfizer and Moderna) of 53.1%, highlighting the importance of a second boost for this risk group [123].

Data regarding the effectiveness against VOIs is still missing, however, the neutralizing activities of sera from fully vaccinated individuals who received BNT162b2 against the Epsilon, Eta, Iota were not significantly diminished in comparison with the ancestral isolate USA-WA1/2020 [113]. Similarly, data disclosed by Moderna showed reduced neutralization for the Eta variant (4.2-fold) in sera from eight fully vaccinated individuals. Additionally, 8-fold reduction in neutralizing titers was reported for A.VOI.V2, a novel variant first identified in Angola. This variant is currently not designated as a VOI despite carrying multiple significant mutations, including T478R and E484K in the RBD, Y144Δ, R246M, SYL247-249Δ in the NTD and P681H adjacent to the furin cleavage site [124]. As of concern, the recent Mu variant seems to be more resistant to antibody neutralization than the Beta variant using sera from BNT162b2-vaccinated individuals, as a preprint study revealed (log_2_PRNT_50_ 90 vs. 76) [125]. These results highlight the urgent need for tailored mRNA.

#### 2.1.5. Adverse Effects Associated with mRNA Vaccination

Vaccination programs have reported cases of local and systemic acute allergic reactions with BNT162b2 and mRNA-1273. A prospective study of 64.900 subjects after a first dose of BNT162b2 (60% of the total cohort) and mRNA-1273 (40%) showed that only 2.1% of the total cohort presented signs of acute allergic reactions like itching or rash, hives or swelling; however, more frequently with mRNA-1273 (2.2%) than with BNT162b2 (1.95%). Anaphylactic events were reported for 16 participants (0.025%) of which 94% were females in average age of 41 years, 63% had previous allergic reactions and 31% had a history of anaphylaxis. It is of note that the incidence rate reported in this study is larger than the incidence reported by the CDC (2.5–11.1 cases per one million doses) [126]. Importantly, cases of rare heart inflammation symptoms have been associated with mRNA vaccines. A published study of seven male individuals below the age of 40 that presented acute onset of chest pain from three to seven days after vaccination, showed evidence for myocardial injury by high or low levels of cardiac troponin I. However, all patients were discharged one to three days post-treatment with beta-blockers and anti-inflammatory medication [127]. In June 2021, the CDC announced a possible link between myocarditis and mRNA vaccination, as the reported cases of heart inflammation post vaccination in the US were higher than the statistically expected values [67]. The Ministry of Health in Israel has also reported this correlation, in which between 1:3000 and 1:6000 men aged 16–24 years developed this rare heart condition.

### 2.2. Adenovirus-Based Vaccines

Adenoviruses have been used as vaccine carriers for several decades and are deployed as vectors against HIV, tuberculosis, malaria and the Ebola virus [128]. They are genetically stable, inexpensive and most importantly, the induced T- and B-cell responses are well characterized [129]. A disadvantage of this technology may be posed by pre-existing host immunity to adenovirus vectors to which the scientific community has expressed its concern with the current SARS-CoV-2 Ad-vaccines [129,130].

#### 2.2.1. AZD1222 (AstraZeneca)

The first vaccine of this technology to be authorized for emergency use in the UK on 30 December 2020, was AZD1222 from AstraZeneca/University of Oxford. Commercialized as Vaxzevria, AZD1222 adopted a replication-deficient chimpanzee adenoviral vector, ChAdOx1. This vector was designed to minimize the ubiquitous seroprevalence in humans and has been utilized for vaccines against MERS-CoV, influenza A viruses (IAV), Zika Virus and Malaria, which has reached phase I/II trials (ClinicalTrials.gov Identifier: NCT03203421) [131,132,133]. The multicenter II/III trial with cohorts in the UK, Brazil and South Africa, demonstrated a 70% overall vaccine efficacy of a two-dose schedule, 28 days apart from each other (ClinicalTrials.gov: NCT04324606, NCT04400838 and NCT04444674). Disease endpoints evaluated in this study were infection severity and reduction of hospitalization, the latter dropping to 59.8% [134]. Post-trial modifications of the original immunization schedule have shown that increasing the time interval between the first and second dose to 12 weeks enhanced the vaccine efficacy to 80% [135]. Moreover, intranasal administration of AZD1222 instead of the intramuscular route has been reported to decrease virus shedding in hamsters and chimpanzees [136].

#### 2.2.2. Sputnik V (Gamaleya Institute)

In comparison to AstraZeneca, the Gam-COVID-Vac (Sputnik V) vaccine from Gamaleya National Research Center for Epidemiology and Microbiology, Moscow, Russia, adopted a combination of two adenoviruses, hAd26 and hAd5 vectors, employed for the first and second shot, respectively, to minimize immune responses against the vectors. Both vectors are administered with an interval of 21 days. The phase III trial demonstrated overall protection of 91.3% for all age (>18 years old) and gender groups tested (ClinicalTrials.gov: NCT04530396). The endpoints of the study were the reduction in host viral load, as tested by PCR, and incidence of severe cases of COVID-19 at day 21 post-priming dose [137]. Immediate reactions to the vaccine were limited to local reactions, with minimal severe events reported. The trial cohort was exclusively from Russia, implying that the vaccine efficacy may vary for other demographic groups. In January 2021, a phase I/II trial was submitted in which a combination of AZD1222 and hAd26 from Gam-COVID-Vac will be tested, which is expected to increase the protection efficiency. First results may be available at the end of 2021 (ClinicalTrials.gov Identifier: NCT04684446).

#### 2.2.3. Ad26.COV.S (Janssen/Johnson & Johnson)

Another adenovirus-based vaccine already approved is Ad26.COV2.S from J&J. This vaccine utilizes an hAd26 vector and, in comparison with other vaccines, follows a one-dose regimen (ENSEMBLE trial, ClinicalTrials.gov: NCT04505722). Vaccine efficacy was calculated at 66.9% in preventing moderate-to-severe forms of COVID-19 and at 76.7% in preventing hospitalization. The US cohort indicated 74% of protection, followed by Brazil with 66% and 52% for South Africa. No severe adverse events were observed in the trial. Interestingly, of the infected participants in Brazil, 70% were infected with the Zeta variant, and in South Africa, 96% of reported infections were by Beta [138]. Currently, the company is running a parallel phase III trial of this vaccine vector using a two-dose regimen to increase efficacy (Study 3009). The first picture of vaccination effectiveness has been published in Finland. Upon the administration of a first dose, effectiveness reached 42% against symptomatic infection and 62% against hospitalization. Effectiveness upon full immunization could not be estimated due to the insufficient incidence of cases [139].

#### 2.2.4. Ad5-nCoV (CanSino Biological Inc.)

Ad5-nCoV (Convidencia), a hAd5 vector vaccine developed by CanSino Biological Inc. (Tianjin, China) was approved for general use on 25 February 2021, by the Chinese government, although their phase III trial data have not been officially published yet. In February 2021, the company indicated a preliminary efficiency of 65.7%, based on a study in Pakistan (ClinicalTrials.gov: NCT04526990). Later, the company reported interim data with an average protection of 68.8%, which dropped to 65.2% after four weeks. The immunization regimen consists of a single dose, which may explain the requirement of a booster. The company has suggested the necessity of a booster shot in order to increase the protection rate.

#### 2.2.5. Effectiveness of Adenovirus-Based Vaccines against Variants

Overall, real-world data suggest an apparent lower effectiveness against symptomatic infection with variants from adenovirus-based than mRNA vaccines. However, regarding effectiveness against severe disease, hospitalization and death, protection seems comparable between technologies. A follow-up study with participants of the multi-centered phase II/III trial showed a preliminary effectiveness of 70.4% in preventing symptomatic COVID-19 following infection with the Alpha variant in this cohort. This multinational study is still ongoing (ClinicalTrials.gov: NCT04400838) [140]. Real-world data from England reports an effectiveness of 74.5% against symptomatic infections by the Alpha variant. With regard to severe disease and hospitalization, effectiveness reached 76% upon the first dose and 86% after the second dose [115]. J&J observed similar antibody-neutralizing activity upon a single-dose of Ad26.COV2.S against this VOC as compared to the parental USA-WA1/2020 [141]. Similarly, Sputnik V full-vaccination induced equivalent neutralization titers against this variant as the reference strain B.1.1.1, locally endemic to Moscow [142].

The effectiveness of the first generation adenovirus-based vaccines against the Beta variant has been reported to be reduced. AZD1222 effectiveness dropped to 21.9% against the Beta variant during the tests in cohorts from South Africa. The neutralization activities of vaccinees’ sera showed a 6-9 fold reduction [143]. However, it is important to point out that the vaccination schedule tested in this study was a 28-day interval, while the three-month interval regimen that is known to induce higher antibody responses was not included in this study. Real-world data from Canada showed that one dose with AZD1222 has a protective effectiveness of 82% against hospitalization or death from infection with Beta/Gamma [116]. Consistently to AZD1222, one-log reduction in the neutralization activity in sera from J&J vaccinated participants in comparison with the reference isolate and with sera from individuals fully vaccinated with Sputnik V, neutralization titers dropped by 3.1-fold [141,142]. This emphasizes the requirement of more specific vaccines against this variant. AstraZeneca has already started its preclinical studies with AZD2816, a ChAdOx1-vectored vaccine that is planned to be used as a booster [144].

For the Delta variant, there is more information available regarding the effectiveness of adenovirus-based vaccines. Serum samples from vaccinated participants with J&J demonstrated similar neutralization levels against USA-WA1/2020 [141]. In a separate study, serum from Ad26.COV2.S vaccinees demonstrated reduced neutralization activity for Ad26.COV2.S against the Delta and Lambda variants, highlighting the benefit of a second dose of Ad26.COV2.S to increase the protection against variants [145]. For serum samples from vaccinated individuals with Sputnik, a statistical reduction was observed of 2.5-fold when compared with the reference isolate [142]. Interestingly, as observed for mRNA vaccines, the neutralizing activity of serum does not always correlate with vaccination effectiveness. The UK has an effectiveness of AZD1222 of 67% against symptomatic infections with this variant and of 71% against severe disease and hospitalization. The latter increased upon the second dose to 92%, according to a retrospective study [115]. This endorses the importance of completing full immunization schedules during mass vaccination programs in order to reach full protection against this VOC. Interestingly, a third dose of AZD1222 more than six months after the second dose induced a strong boost to immunity against SARS-CoV-2, including the most common variants: Alpha, Beta and Delta [146]. On 30 June 2021, the director of Moscow’s Gamaleya Institute communicated, in a press conference, an estimation of 90% of effectiveness of the vaccination with Sputnik V in Russia, calculated based on digital medical and vaccine records. However, no official data have been disclosed [142]. In cases of other variants, the neutralization activity of serum from subjects vaccinated with Sputnik V demonstrated no significant differences between neutralization titers for the Delta sublineage B.1.617.3 and Moscow endemic lineages, B.1.1.141 and B.1.1.317. In general, Sputnik V appears to be effective against variants; however, transparent data is still missing.

#### 2.2.6. Adverse Effects Associated with Adenovirus-Based Vaccines

By mid-March 2021, vaccination with AZD1222 was stopped in several countries due to increased cases of embolism and thrombosis that were possibly associated with this vaccine. From almost 24 million first doses, 256 cases of thrombosis were reported worldwide, of which 45 were fatal. Of 30 cases in the EU, 19 occurred in women and 11 in men. Manifestations were deep vein thrombosis, hepatic vein thrombosis, mesenteric vein thrombosis, portal vein thrombosis pulmonary embolism, thrombocytopenia, disseminated intravascular coagulation, among others. The symptoms differentially appeared over a time span of up to 16 days post-immunization. After investigation of cases with venous thromboembolism following vaccination, the European Medicines Agency (EMA) concluded that these disorders occur naturally, in all age groups, and are not uncommon. Given the 24 million vaccine applications, the incidence was considered low and the evidence was insufficient to establish a causal association to the vaccine. In accordance with this, a report from Denmark demonstrated that the incidence of venous thromboembolic events from 2010 to 2018 has a higher incidence than the cases reported along with AZD1222 vaccination, supporting the conclusion reached by EMA [147]. Several studies pointed out the resemblance of heparin-induced thrombocytopenia (HIT) to the thrombotic disorders observed in vaccinees, although heparin is not included in the formulation of AZD1222. HIT occurs during heparin treatment when human platelet factor 4 (PF4) interacts with heparin-forming complexes that trigger the production of PF4-heparin-specific antibodies. These antibodies recognize complexes of PF4 and cell-surface glycosaminoglycans (PF4-GAG) on platelets and monocytes leading to cell activation and thrombocytopenic effects [148]. However, HIT can also be triggered in the absence of heparin exposure, which is denominated as immune heparin-induced thrombocytopenia (HIIT). In Germany, a study of 11 patients vaccinated with AZD1222 demonstrated high levels of antibodies specific for PF4-heparin complexes and PF4-dependent platelet activation in sera samples [149]. Comparable results were reported in the UK and Norway [150,151]. This PF4-dependent thrombotic disorder is now referred to as vaccine-induced immune thrombotic thrombocytopenia (VIIT) and is characterized by thrombosis in unusual regions of cerebral sinuous veins and lung or splanchnic veins with mild-to-severe thrombocytopenia. Structural studies of ChAdOx1 suggested that the electronegative potential of the viral capsid could lead to interaction with PF4, leading to complex formations and antibody production, as observed in HIIT [152]. The probability to develop VIIT has been calculated to be one per 250,000 vaccinees. Compared to the risk to develop HIIT due to oral contraceptives or air travel, which accumulates in 1:1000 and 1:2000 people, respectively, the risk to develop VIIT seems low [153]. In the middle of May 2021, EMA updated the product information of AZD1222 adding VIIT as a rare side effect. EMA also recommended that health personnel must not proceed with a second dose if a person has presented blood cloths or low platelet numbers after vaccination and clinicians should monitor platelet levels up to three weeks after vaccination.

Similar VIIT disorders have been reported for vaccination with Ad26.COV2.S in the US, leading to a pause in the vaccination program with this vaccine in the middle of April 2021 [154]. Of note, 28 VIIT cases have been reviewed by the CDC and the FDA out of more than eight million doses applied at the time of the evaluation. Given the low incidence and the insufficient evidence, the Advisory Committee on Immunization Practices (ACIP) and CDC resumed vaccination with Ad26.COV2.S among people 18-years and older. One study also reported VIIT cases after vaccination with the mRNA vaccines BNT162b2 and mRNA-1273 [155].

#### 2.2.7. Real-World Adaptions: Heterologous Vaccination

In response to the stop of vaccination with adenovirus-based vaccines, young women who had received a first dose of these vaccines were advised to use mRNA technology for the second dose although the lack of information in clinical studies. Today, available results from studies investigating the safety and efficacy of heterologous vaccination series are very encouraging. Preliminary studies conducted in animal models demonstrated that a heterologous vaccination schedule with an adenovirus-based vaccine as a prime dose followed by an mRNA vaccine as a booster dose is associated with increased levels of neutralizing antibodies and improved Th1-based T cell responses [156]. The British Com-COV study (Comparing Covid-19 Vaccine Combination Schedules, ISRCTN 69254139), tests both, AZD1222 and BNT162b2 vaccines, in different order to compare all four prime-boost permutations at different intervals (28-day and 84-day intervals). First interim analysis of the Com-COV study (published May 12th, 2021) evaluated reactogenicity and immunogenicity. Compared to homologous vaccination schedules, the frequency of mild and moderate vaccination reactions was increased in participants who received a heterologous prime-boost with AZD1222/BNT162b2. Most reactions occurred within 48 h of immunization, but hospital admissions were not required [157]. The immune response of AZD1222/BNT162b2, measured by the geometric mean of SARS-CoV-2 anti-spike IgG antibody concentrations (GMC, Geometric Mean Concentration), was significantly stronger at 28 days (12906 ELU/mL) than with AZD1222/AZD1222 (1392 ELU/mL). Similarly, the T cell immune response of the heterologous vaccination regimen was more pronounced than that of the homologous vaccination regimens [158]. These results indicate that the immune response achieved after heterologous vaccination regimen is significantly stronger and most likely, due to the significantly higher antibody concentrations, also more durable than the immune response after a homologous vaccination series. At present, up to fifteen countries (Austria, Bulgaria, Czechia, Denmark, Finland, France, Germany, Iceland, Italy, Luxembourg, the Netherlands, Norway, Portugal, Slovenia and Spain) are using a heterologous vaccination regimen [159]. As the combination of AZD1222 and mRNA vaccines has been shown to elicit a robust humoral response against SARS-CoV-2 and higher T cell responses than homologous combinations, in Germany the vaccine advisory committee (STIKO) already recommends a heterologous vaccination schedule for all individuals who have received AZD1222 as the first dose and an mRNA vaccine as the second dose, with a vaccination interval of at least four weeks to the first dose [160].

### 2.3. Inactivated Virus and Recombinant Protein Vaccines

The usage of chemically inactivated viruses is the most traditional approach for vaccine development and is suggested to offer a broader range of native-conformation antigens compared to spike-only vaccines. All prototypes are based on coronavirus isolates from hospitalized patients in the early pandemic phase (lineage A). Pharmaceutical companies of China lead this technology and their phase III trials, with different cohorts worldwide, are still ongoing. However, official data has not been published in peer-reviewed journals and, similarly, vaccination-effectiveness data is still missing, even though WHO and other international entities have recommended and authorized their use.

#### 2.3.1. CoronaVac (Sinovac Biotech)

Sinovac Biotech started its clinical assessments on 16 April 2020. Their phase III trial is still ongoing in Brazil, the Philippines, Chile, Indonesia and Hong Kong. Recently reported intermediate results from the cohort in Turkey (ClinicalTrials.gov: NCT04942405) showed a vaccine efficacy of 83.5% in a two-dose regimen with a 14-day interval in preventing symptomatic disease, confirmed by RT-PCR. These results are limited to age groups between 18 and 59 years old [161]. Press release from the company has announced preliminary efficacy of 50.6% in reducing symptomatic COVID-19 in the cohort of Brazil (ClinicalTrials.gov: NCT04456595). These large discrepancies between cohorts might be the product of divergent study design as well as differences in the prevalence of circulating SARS-CoV-2 variants. In June 2021, WHO has validated CoronaVac for emergency use and issued its recommendation.

#### 2.3.2. BBIBP/ WIV04/HB02 (Sinopharm)

Sinopharm collaborated with the Beijing Institute of Biological Products to create an inactivated vaccine named BBIBP-CorV [162]. The phase III study utilizes a two-dose regimen with three weeks interval and is currently underway in cohorts from different continents including Argentina, Peru, United Arab Emirates, Bahrain, Egypt and Jordan (ClinicalTrials.gov: NCT04510207, NCT04560881, NCT04612972, ChiCTR2000034780). WHO advisory board announced an estimated efficacy of 78.1% from their multicenter cohorts. Yet, final data has not been disclosed. In parallel, the company teamed up with the Wuhan Institute of Biological Products to create two vaccine prospects that were evaluated hand-in-hand in the same phase III trial. These two vaccines were derived from the WHO-certified viral isolates, WIV04 and HB02, respectively [163]. Their phase III trial demonstrated that a two-dose schedule, 21 days apart, offers protection of 72.8% with WIV04 vaccine and 78.1% with HB02 against moderate-to-severe forms of COVID-19. No severe adverse effects were reported for both vaccine prospects during the trial. COVID-19 cases in Seychelles, a country with a high Sinopharm vaccination rollout, started to increase once the borders were reopened to tourism. Among the positive cases, 37% were from fully immunized subjects. However, no fatal cases were observed among the vaccinated infected cases.

#### 2.3.3. BBV152 (Bharat Biotech)

In an effort of the Indian Council of Medical Research together with the National Institute of Virology, the Indian company Bharat Biotech created BBV152 (also known as Covaxin). This vaccine obtained the EUA in India on 3 January 2021. The NIV-2020-770 vaccine isolate that presents the D614G mutation is formulated with imidazoquinoline absorbed in Alum-gel (Algel) instead of aluminum hydroxide [164]. The published results of its phase III trial report an overall efficacy of 77.8% against symptomatic disease, 93.4% against severe disease and hospitalization using a vaccination scheme of two injections with an interval of 28 days within the cohort in India (ClinicalTrials.gov: NCT04641481) [165]. An efficacy of 63.6% against asymptomatic infection was estimated by laboratory-confirmed viral load. Moreover, the company reported an estimated efficacy of 63.6% against the Delta and 90.1% against the Kappa variant, *ad hoc*, by implementing the sequencing of nasal samples from the already enrolled participants, as these variants emerged during this study. Real-world data regarding effectiveness of Covaxin is still awaited.

#### 2.3.4. NVX-CoV2373 (Novavax)

Novavax created a recombinant nanoparticle vaccine NVX-CoV2373. The full-length spike protein is produced in an insect cell-expression system and is combined with saponin-based Matrix-M as adjuvant. Mechanistically, NVX-CoV2373 elicits receptor blocking, virus-neutralizing antibodies and Fc-effector functional antibodies when investigated in non-human primates [166]. Results of their phase III trial in the UK demonstrated an overall efficacy of 89.7% in reducing mild-to-severe disease forms. This efficacy was observed seven days upon a second dose, using a two-dose regimen, 21 days apart. Of note, 27.9% of total participants were above65 years of age [167]. The company announced the first numbers regarding the ongoing phase III trial in the US and Mexico (ClinicalTrials.gov: NCT04611802) in a press release. Similar vaccine efficacy as in the UK, 90.4%, has been reported in the group of 18–65 years old and 91% in the high-risk group of those >65 years old. Novavax has already filed for approval to the EMA for European distribution of its already purchased 100 million doses at the end of 2021.

From the reviewed data it may appear as if mRNA vaccines that reach up to 90% effectiveness are superior to the other vaccines, but real-world data displayed high effectiveness and are able to reduce the high risk of developing severe COVID-19. In comparison with other vaccines against respiratory viruses e.g., seasonal influenza, which present effectiveness ranging from 40–60% or 39% for respiratory syncytial virus infections, all SARS-CoV-2 vaccines have been demonstrated to be extraordinary powerful [168,169].

There are several factors that affect the effectiveness of all vaccine types, such as age, the number of boosters administered and the duration between them. In general, one-dose vaccines like Ad26.COV.S or Ad-nCoV seem to be less efficacious than two-dose vaccines, highlighting the importance of booster doses in order to maximize humoral immune responses. This is also reflected in studies determining neutralizing antibody titers in the blood of individual after the first and the second dose. Elderly persons, over 70 years old, have been observed to remain vulnerable to SARS-CoV-2 infections, as real-world data has reported a clearly reduced effectiveness of 56%, pointing out the importance of second booster doses in this risk group to retain good protection. The future will tell whether there are differences in the duration of the protective immune responses, especially in the induction of mucosal protection. However, since heterologous vaccine combinations will most likely become normality, these differences will become less important. An important limitation of all first-generation SARS-CoV-2 vaccines is the lack of information regarding asymptomatic infection. This type of infection was not evaluated in phase III trials giving room for speculation about vaccine effectiveness and virus shedding among vaccinees. This important aspect remains still to be addressed in further epidemiological studies.

## 3. Real-World Needs and Expansion of Clinical Investigations to Vulnerable Groups

### 3.1. Vaccination of Pregnant Women

Although there have been controversial reports as to whether pregnant women are more susceptible to SARS-CoV-2 infections, a recent multinational cohort study in 18 countries found that pregnant women with COVID-19 diagnosis are at higher risk of maternal complications, pre-term birth and loss of the fetus [170]. In recognition of this special vulnerable group, Biontech/Pfizer started a phase II/III trial on pregnant women, applying the same vaccination schedule as used for their trials in adults: 30 µg in a two-dose schedule in a cohort of 4000 healthy pregnant women at 24 to 34 weeks gestation. First results are planned to be reported on June 2022 (ClinicalTrials.gov Identifier: NCT04754594).

Prospective studies in the US have presented data regarding the safety of the first-generation vaccines BNT162b2 and mRNA-1273 in pregnant women. A first study of 131 participants, in which women were monitored from their first vaccination dose with either BNT16b2 (50% of the cohort) or mRNA-1273 (50%) until delivery, demonstrated that mRNA vaccination is safe, as pregnant women presented similar reactogenicity as non-pregnant women. Moreover, evaluation of humoral responses induced upon partial vaccination demonstrated that blood levels of IgG, IgA and IgM antibodies are equivalent to those in the non-pregnant group. Of interest, neutralizing antibodies were also found in umbilical cord and breastmilk. Upon full vaccination, IgG titers increased further in the maternal and breastmilk, while IgA titers were not modified [171]. These findings indicate high vaccine efficacy and safety for pregnant women as well as transmission of neutralizing antibodies from mother to fetus. Consistently, results from the “V-safe” program, a follow-up, smartphone-based program developed for surveillance of the vaccination program, demonstrated similar findings regarding reactogenicity of vaccination in pregnant women. Spontaneous abortion and stillbirth were reported more frequently in women who got the first immunization dose in the first trimester (92.3%) than in the last trimester [172]. However, whether this is associated with vaccination requires more investigation.

### 3.2. Vaccination of Children Aged <15 Years

There is an ongoing debate on the level of susceptibility of children to SARS-CoV-2 infection and the development of COVID-19 or other related diseases and how this group shapes the dynamic of the pandemic. However, as children are developing mild and severe COVID-19 as well as long-term symptoms with diverse manifestations and social distancing practices that are difficult to apply for the youngest, there is a need for safe and efficient vaccines for children. Biontech/Pfizer has published results from their phase I/II/III trial in children from 12 to 15 years old. An overall vaccine efficiency of 100% is reported seven days upon second dose application. Of note, immunogenicity in this group was higher compared to the control group of those 16 to 25 years old. Local and systemic reactions due to vaccination are observed at similar frequency as in the group control. This demonstrates that BNT162b2 is safe for adolescents as well as children over 12 years old [173]. In comparison, clinical trials in children under 11 years are planned to investigate the scale-down of dosing according to age. Investigators have started these trials using 10 µg instead of 30 µg dose, presuming that immune systems of the young are more reactive.

Moderna released similar preliminary results from their study “TeenCOVE” (ClinicalTrials.gov: NCT04649151). Using a cohort of more than 3000 children between 12 and 17 years old, demonstrated a vaccine efficacy of 93% against mild disease, given the low frequency of severe disease among adolescents. Disease was confirmed by quantification of the viral load by RT-PCR 14 days after the first dose using 100 µg of mRNA-1273, similar to the dosage used for adults. The phase I/II/III clinical trial in children under 11 years is already ongoing.

### 3.3. Mortality in Aged Care Facilities (ACF)

COVID-19 outbreaks in aged-care facilities (ACF) are specifically devastating. Pre-vaccination mortality rates have been estimated to be over 40% in countries like the US or UK, or even higher than 70%, as observed in Australia and Canada [174]. Despite the high prioritization and high rates of vaccinated residents, outbreaks and death inside ACFs are continuously reported. This may be partially explained by the reduced vaccine effectiveness observed among the elderly, given the lower immunogenicity triggered by the vaccines in this group [175], as well as the reintroduction of infections by unvaccinated staff or visitors. A retrospective study in Denmark from almost 40,000 participants of 84 years old on average demonstrated that there was no significant vaccine effectiveness conferred by the first vaccine dose. However, it increased to 52% during the first seven days after the second dose and, later, to 64%. It is of note that 99% of the subjects were vaccinated with BNT162b2 [176]. As a reaction, and against the WHO recommendations to provide vaccine doses to third world countries to increase the number of individuals with one vaccine dose, several countries, such as Israel, Russia and Hungary, have started to offer third vaccine doses (second boosters) for adults at higher risk of severe COVID-19, including the elderly, as well as individuals with a weak immune system and other comorbidities. Other countries, such as the UK, France or Germany, are planning to administer third doses starting September 2021.

## 4. Real-World Challenges for SARS-CoV-2 Vaccines

### 4.1. Delaying the Second Dose

During COVID-19 vaccine shortages, the question arose as to how the available doses could be distributed most effectively in order to reach fast and broad protection within the population. Some countries, such as the UK or Canada, have adapted the recommended vaccination regimen to rapidly increase the number of individuals who have received at least one vaccine dose and prevent severe disease on a broader scale. The UK Medicines and Healthcare Products Regulatory Agency (MHRA) decided to increase the interval of the BNT162b2 vaccine prime and boost to up to 12 weeks instead of 21–28 days [177,178]. This approach was rationalized with calculations of data from clinical trials demonstrating that the first dose of the mRNA vaccines BNT162b2 and mRNA-1273 already reached an efficacy of 92.6% and 92.1% [104,179].

Recently, new research suggests that longer intervals are a successful strategy, regardless of the type of vaccine. Based on a mathematical model comparing the epidemiological impact of different vaccination strategies, it was shown that delaying the second dose of BNT162b2 and mRNA-1273 (9–15 weeks after the first dose) could reduce new infections, hospitalizations and deaths in countries with limited vaccine supply and distribution capacity [180]. This strategy is corroborated by data showing that older people (>80 years old) had a 3.5-fold higher antibody response if the second BNT162b2 vaccine was delayed to 12 weeks, rather than three weeks [181]. A delayed second dose of AZD1222 also resulted in a stronger immune response, as people who received their second dose at 44 to 45 weeks had higher antibody levels than those with an 8- to 12-week interval [146].

Real-world developments currently show a strong increase in infection numbers, as well as the incidence of mild-to-severe disease, even in vaccinees. However, the numbers of hospitalizations and deaths are still low. In Israel, which has the highest number of fully vaccinated individuals using the BNT162b2 vaccine, steadily increasing numbers of infections are being recorded. Since June 2021, the Israel Ministry of Health has observed, simultaneously with the spread of the Delta variant, a decline in the effectiveness of BNT162b2 in preventing infection and symptomatic illness (both 64%) [182]. Based on the decreasing protective effect of BNT162b2 against infection and symptomatic diseases shown in Israel, a second booster dose might therefore be necessary 6 to 12 months after the first booster [183]. Whether or not to delay the second dose is an ethical issue that is being debated worldwide. The underlying idea is to prevent deaths by delaying a second injection that would temporarily release the vaccine to twice as many people. Therefore, in this view preventing more people from death is a priority. However, countries are reconsidering this scheme due to the observed increased protection of the second dose against Delta-variant infections, which is currently the dominant variant worldwide. Nevertheless, authorities are urged to be transparent about their decisions in order to allow the public to understand the arguments and thus maintain confidence in vaccination.

### 4.2. Vaccination and Long COVID

Long-lasting health damage following SARS-CoV-2 infection, commonly termed as “long COVID” or “post-COVID-19 syndrome”, has finally been recognized as a global public health problem. Patients suffering from long COVID report a variety of symptoms long after recovery, regardless of severity of initial infections, including fatigue, cough, chest tightness, shortness of breath or myalgia [184]. So far, therapeutic options for long COVID are not available. Interestingly, a not-yet-peer-reviewed prospective observational study observed that administration of SARS-CoV-2 vaccines can improve long COVID symptoms in people who had been hospitalized with acute COVID-19 [185]. In this study, patients reported numerous symptoms, among them, fatigue (61%), shortness of breath (50%) and insomnia (38%) were the most common. However, patients noticed an improvement of these symptoms at a median of 32 days after vaccination with either the Biontech/Pfizer or AstraZeneca vaccines as compared with unvaccinated participants (23.7% vaccinated vs. 15.4% unvaccinated).

A scientifically accompanied survey provided further real-world insights into the effects of SARS-CoV-2 vaccination on long COVID [186]. In the survey, about 900 people, with past SARS-CoV-2 infection and at least one vaccine dose from AstraZeneca (50%), Biontech/Pfizer (40%), Moderna (8.6%) or J&J (1%) were asked to rate their symptoms before and after vaccination. This revealed that 57% of the participants experienced an overall reduction in their symptoms, while 24% reported no difference and 18.7% experienced a deterioration. In general, the mRNA vaccines, especially the Moderna vaccine, appeared to be more advantageous in terms of symptom improvement, with an average reduction of the symptoms of 31% (Moderna) and 24.4% (Biontech/Pfizer).

A possible explanation for the effect of vaccination on long COVID symptoms was recently suggested in an as yet non-peer-reviewed study [187]. In the study, the authors found that SARS-CoV-2 infection leads to the generation of dysfunctional and potentially pathogenic, double-negative memory B cells. However, after vaccination, the frequency of these cells was decreased. These results should be encouraging to those around the world who have developed persistent symptoms of illness after coronavirus infection, and their considerations of whether to become vaccinated or not. Additionally, we have learned that long COVID is not only problematic for subjects that presented severe infections or hospitalization, but also in young, home-isolated patients with mild infections [188]. However, more studies are required to understand how infections lead to long COVID and how it impacts the life quality.

### 4.3. Breakthrough Infections

Although two doses of the most widely used mRNA-based SARS-CoV-2 vaccine BNT162b2 provide 95% protection against symptomatic COVID-19, none of the approved vaccines shows an efficacy of 100% [104]. SARS-CoV-2 infections diagnosed in fully vaccinated people (at least two weeks after two doses of BNT162b2, mRNA-1273, AZD1222 or one dose of Ad26.COV2.S) are known as breakthrough infections. However, the majority of breakthrough cases have been reported as mild or asymptomatic and were not associated with hospitalization or death. As of 19 July 2021, among the more than 161 million vaccinated people in the US, the CDC reported a total of 5914 breakthrough infections in which patients were hospitalized or died. Of note, three-quarters of these cases occurred in people over the age of 65, suggesting that there are certain risk groups among the vaccinated [189]. Also in Germany, 6125 vaccine breakthrough infections have been identified since the beginning of February 2021, of which 0% were hospitalized at age <18 years, 2% at age 18–59 years and 27% at age ≥60 years [190]. In addition, comorbidities and immunosuppression appear to increase the risk of breakthrough infection and a more severe course of illness, as recently shown in Israel. Only six of 152 patients who had to be hospitalized despite being fully vaccinate, were previously healthy. The other patients had been diagnosed with multiple co-morbidities, including hypertension, diabetes, congestive heart failure, chronic kidney and lung diseases, dementia and cancer [191]. Furthermore, there is also evidence that variants will cause some vaccine breakthrough cases. Recently, two women with vaccine breakthrough infections were identified in a cohort of 417 employees of the Rockefeller University who were vaccinated with BNT162b2 or mRNA-1273 [192]. Clinical symptoms of COVID-19 were observed 19 days and 36 days after receiving the second dose of the vaccine. Sequencing of the virus revealed that both women were infected with SARS-CoV-2 variants. Compared with the original sequence first identified in Wuhan, China, viral sequencing revealed several differences in these variant viruses, including mutations in the spike protein including E484K and D614G and mutations of D614G and S477N, respectively. In one patient, the variant strain was found to be related to but still distinct from the Alpha and the Iota variants. Data from another study, in which participants were immunized with BNT162b2 and tested positive for SARS-CoV-2 between 14 days after the first dose and 7 days after the second dose, suggesting that the Alpha variant is more effective at breaking immunization in individuals with only one shot than the reference isolate. The same was found for the Beta variant [193].

## 5. Conclusions

The fast development of safe and highly effective vaccines against SARS-CoV-2 infections, including the first broad use of mRNA-based vaccines, is a real success story and an impressive demonstration of the power of medical and basic research. Even today, new vaccine approaches are underway, giving rise to 300 vaccine candidates, of which more than 100 are under clinical investigation [194]. To date, more than 5.5 billion vaccine doses have been administered globally, as reported by the WHO on 14 September 2021 [88]. More recently, very promising progress has been made in the evaluation of the vaccines in pregnant and lactating women, demonstrating that mRNA vaccines are safe and well-tolerated for mother and child within this particularly vulnerable group. With respect to the proposed risks to mother and child posed by natural SARS-CoV-2 infections [195], this development is a huge success. Similarly, vaccination of younger children, even without immune-compromising comorbidities, is already on the way to being recommended more globally, although the vulnerability to infection, COVID-19 and long COVID of this group has been a controversial issue and is still under intense investigation. However, the possibility for parents to decide to vaccinate their children is an important improvement.

Despite this success, the aim of reaching global herd immunity through vaccination, which would allow the abandonment of other non-pharmaceutical interventions and the restrictions of our social, cultural and recreational activities has not yet been reached in every country. This is due to a number of reasons and unexpected developments that have challenged and delayed vaccination progress.

One of the most impactful developments has been the rapid local and global emergence of SARS-CoV-2 variants with different transmission and immune evasion properties. This has not only initiated new waves of infections but also impacted the evaluation of vaccine efficacies in clinical trials, as we have summarized in this review. To provide rapid estimations on the potential loss of protection by the vaccines against the variants many studies have investigated neutralizing activities in the serum of vaccinated individuals using diverse assays, employing either whole virus, VSV-pseudotyped particles carrying spike proteins with the respective mutations or recombinant protein assays such as ELISA, that rely on either the entire spike protein or just the RBD. Naturally, such in vitro assays are the fasted available tools for this purpose when compared to time-consuming in vivo studies. However, it needs to be communicated clearly that they can only partially assess the quality of the complex protective immune responses to SARS-CoV-2. As summarized in this review, several of these reports have indeed indicated a partial reduction in the neutralizing activity against several variants. However, it is currently unknown how the neutralizing titers determined in these assays correlate to the protection from infection, severe COVID-19 and death. While this is not of such great concern for the scientific community because the overall effectiveness of the vaccines to prevent severe COVID-19, hospitalizations and death from SARS-CoV-2 infections still remains remarkably high, as observed in all countries with high vaccination rates, the reports on rare complications, such as myocarditis and sinus vein thrombosis as well as reduced vaccine efficacies seem to negatively affect the vaccine acceptance in the broader public. This is evident by a rising number of individuals questioning the personal and social benefits of vaccination, since infections are not fully prevented. Despite huge efforts to close this communication gap by scientists and politicians, in some countries, e.g., Germany (vaccination rate: 66%), this discussion has contributed to a worrying deceleration of progression in vaccination rates despite access to free-of-charge vaccine doses.

Another major hurdle to global immunity is posed by a strong imbalance in world-wide vaccine distribution. While, by now, high-income countries have reached vaccination rates of up to 90%, such as seen in the United Arab Emirates (90%, including single and double doses), Portugal (87%) and Qatar (81%) and are starting to resume normal life, developing and low-income countries that suffer from a lack of scientific infrastructure, as well as their own vaccine production facilities are still undersupplied with vaccines and at high risk of experiencing devastating pandemic waves [88]. Naturally, this imbalance fosters the emergence of SARS-CoV-2 variants, as the virus, in such circumstances, rages unrestricted, with high infection rates through whole populations that will, sooner or later, also be distributed globally. That this is not some scary-movie scenario is currently demonstrated by the emergence and dominant global spread of the Delta variant; this short-sighted global strategy to fight the SARS-CoV-2 pandemic will likely result in a long-term arms-race, necessitating the frequent adaption of vaccines and will, beyond its economic expense, also cost a high number of lives. From ethical, epidemiological as well as economical standpoints, a successful and humane strategy to overcome a viral pandemic, as we are experiencing it today, must be focused on the prevention of infections, severe disease and death on a global level [196]. A concerted global pandemic strategy will minimize the burden for poor but also for wealthy countries and relies on the equal distribution of vaccines to establish broad-scale immunity on a global level. This must be enforced and actively conducted by the nations that in charge of the prospering vaccine production sites.

To improve vaccine efficacies and boost mucosal immunity against current and future SARS-CoV-2 variants researchers and vaccine companies are already investing the development of second-generation vaccines using diverse strategies. Interestingly, studies have shown that, in contrast to the early Wuhan-1 virus isolate or the Alpha variant, antibodies elicited in response to the Beta variant exhibit potent cross-reactivity as demonstrated by the binding and neutralization of S proteins from the Beta and P.1 variants, which emphasizes use of the Beta variant spike as a seed antigen for the next generation of vaccines [197,198]. Hence, Biontech, CureVac and AstraZeneca have designed a second-generation vaccine based on the Beta-variant S protein mutations. Prospectively, the second-generation vaccine from Astra Zeneca and collaborators (AZD2816), which is, again, ChAdOx1 vector-based, could be used as a second booster to enhance protection against the Delta variant. Preclinical studies in mice have shown that AZD2816 can enhance antibody responses against the Beta variant and provide protection against both the Kappa and Delta variants [144]. Further improvements may be achieved by the introduction of mutations that affect S protein topology, such as mutation T343A, which changes the NTD glycan shield and contributes to stabilization of the RBD in the UP-position [199] or the incorporation of motifs that support S protein trimerization [79]. In parallel to the development of VOC-matched vaccines based on the approved vaccines, which is the most straight forward approach as they are easy to produce and will only require basic clinical re-evaluation, studies to explore the concept of a pan-Corona vaccine that can provide protection across all variants, as well as intranasally applied vaccines to boost local mucosal immune responses are being investigated [200,201,202,203].

In addition to the remarkable scientific achievements, this pandemic has given rise to many additional questions and discussions. Beyond the broad discussions on the origin of SARS-CoV-2, much attention has also been drawn to the consequences of our social and economic behaviors that contribute to the ongoing exploitation of unique ecological niches and thereby foster the emergence of new zoonoses, some of which may have pandemic potential. While these aspects, as well as the growing risks of emerging pathogens that call for improved strategies of pathogen surveillance and pandemic preparedness, have been long communicated by the scientific community over many years, it seems as if this pandemic was needed to bring them to the attention of the general public and political decision-makers. With the novel mRNA vaccine technology, the time frame for generating a vaccine against a new pathogen has been substantially decreased. However, the success of the SARS-CoV-2 mRNA vaccines have also strongly relied on long-term knowledge on the high antigenicity of the S protein, gained from previous coronavirus outbreaks and years of basic research. That such developments require stable and significant investments into basic and medical sciences must be included in the current and future discussions on how we can prepare and protect the world population from further pandemics.
